# War and Peas: Molecular Bases of Resistance to Powdery Mildew in Pea (*Pisum sativum* L.) and Other Legumes

**DOI:** 10.3390/plants11030339

**Published:** 2022-01-27

**Authors:** Anton S. Sulima, Vladimir A. Zhukov

**Affiliations:** All-Russia Research Institute for Agricultural Microbiology (ARRIAM), Laboratory of Genetics of Plant-Microbe Interactions, Podbelsky Sh. 3, 196608 Saint-Petersburg, Russia; vzhukov@arriam.ru

**Keywords:** powdery mildew, Fabaceae, *Pisum sativum*, *MLO* gene family, *er1*

## Abstract

Grain legumes, or pulses, have many beneficial properties that make them potentially attractive to agriculture. However, the large-scale cultivation of legumes faces a number of difficulties, in particular the vulnerability of the currently available cultivars to various diseases that significantly impair yields and seed quality. One of the most dangerous legume pathogens is powdery mildew (a common name for parasitic fungi of the order Erisyphales). This review examines the methods of controlling powdery mildew that are used in modern practice, including fungicides and biological agents. Special attention is paid to the plant genetic mechanisms of resistance, which are the most durable, universal and environmentally friendly. The most studied legume plant in this regard is the garden pea (*Pisum sativum* L.), which possesses naturally occurring resistance conferred by mutations in the gene *MLO1* (*Er1*), for which we list here all the known resistant alleles, including *er1-12* discovered by the authors of this review. Recent achievements in the genetics of resistance to powdery mildew in other legumes and prospects for the introduction of this resistance into other agriculturally important legume species are also discussed.

## 1. Introduction

Legumes (Fabaceae) are the third largest land-plant family, accounting for about 7% of flowering plant species [1]. Grain legumes, or pulses, have long been an important human and animal food source, providing protein, carbohydrates, and fiber [2]. Moreover, they are known for their ability to consume atmospheric nitrogen through symbiosis with rhizobia [3], leading to an increase in soil fertility that enables a reduction in the use of chemical nitrogen fertilizers, which significantly contribute to the greenhouse effect [4]. Despite this, domesticated legumes still occupy a minimal part of arable land, which is mostly dominated by cereal crops; soybeans, the most cultivated legume in the world, are still far below the major cereals (e.g., rice, wheat, maize) [5]. As the ever-growing human population faces global challenges such as the risk of climate change and the increasing demand for food and energy, the usage of environmentally friendly pulse crops has become more and more prominent, though still not equally worldwide. While the introduction of legumes into agricultural systems can, among others, reduce the risks of monoculture, disrupt pathogens’ life cycles and promote soil biodiversity; the benefits of such crop diversification do not often deliver immediate and/or apparent financial profits. In addition, pulse crops, having been neglected en masse for a long time, are currently inferior to cereals in terms of the quantity and quality of the available cultivars, and their genetic resources remain largely unexplored and unexploited [2,6,7]. These include the resistance to their own pathogens and pests [8,9], among which one of the most notorious and dangerous are the so-called powdery mildews, a group of parasitic fungi from the order Erysiphales [10,11,12]. Under suitable conditions, powdery mildew is able to cause up to a 50% loss in the yield of legumes such peas or soybeans [13]. In this review, we discuss the chemical and biological methods of controlling powdery mildew, examine the natural resistance to powdery mildew in the important crop legumes, especially in the garden pea (*Pisum sativum* L.) as the main and best-studied example of natural *mlo1*-based recessively inherited resistance [14,15], and list the main advances that have been made in studying and applying the molecular-genetic bases of this type of host–parasite interaction.

## 2. Know Your Enemy

### 2.1. The Causative Agent of Powdery-Mildew Disease

Powdery mildews are ascomycete fungi of the order Erysiphales (family Erysiphaceae). They are obligate biotrophs of worldwide distribution, presently including 16 genera with approximately 900 species [16,17]. All are parasites of vascular plants, predominantly dicotyledons, of which they can infect about 10,000 species [12,16,17]. Unlike most plant pathogen fungi, which grow within plant tissue, powdery mildews live mostly epiphytically, forming mats and fields of whitish hyphae on almost any plant organ. While different powdery mildews have been used to study key aspects of host–parasite interactions, developmental morphology, cytology, and molecular biology [18,19,20,21,22,23], there are still many unanswered questions, as recent research has shown that the biology of powdery mildews is more complex than previously realized, and their systematics still undergo major revisions [24,25,26,27].

The life cycle of powdery mildew can involve both sexual and asexual stages (teleomorph and anamorph, respectively), or lack either of them [28,29]; in some species, the teleomorphic stage develops depending on weather conditions, namely the level of moisture [12,30]. Life cycles are usually synchronized with those of the host plants, so in order to prevent or control the disease, an understanding of how a particular host–pathogen system functions in a particular environment is required [31].

**Figure 1:**
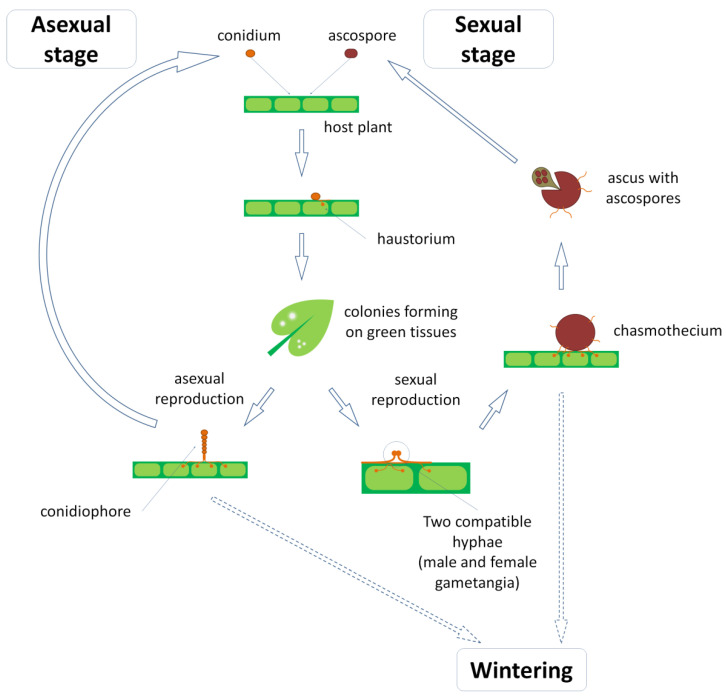
General scheme of powdery-mildew life cycle. Details are explained in text.

There are three main stages in the powdery-mildew life cycle: infection, reproduction and perennation (wintering) (Figure 1). An infection is initiated when an ascospore or conidium lands on a susceptible host and germinates to form a hypha with appressoria, which are short, lateral hyphal outgrowths or swellings producing penetration pegs to infect the host [32,33,34]. Plants can interrupt the germination of spores with so-called pre-penetration defense mechanisms [9,35]. These mechanisms remain poorly understood, but it is known that the physical structure and chemical composition of the host surface, especially of epicuticular leaf waxes, suppress the germination and appressoria formation of powdery mildew [36,37].

Penetration pegs formed by appressoria are narrow protrusions that pierce the walls of host cells. The haustorium is an extension of the penetration peg within the host cell that serves as an interface between parasite and host, causing the plant to divert nutrients to the fungus [32]. The most efficient and well-known resistance mechanisms against powdery mildews are those that efficiently impede the penetration peg to breach plant cell wall [38,39]. These mechanisms provide durable and broad-spectrum resistance to biotrophic pathogens including powdery mildew, which are usually associated with the *MLO* gene family (see below). Plants can also modify their transporter systems to relocate sugars away from infected cells, thus impeding fungus development [40,41]. Orthologs of the *STP13* (Sugar Transport Protein 13) subfamily seem to play a key role in this mechanism.

After the infection takes hold, the hyphae elongate and branch, forming circular colonies. Upon the anchoring of fungus to the host tissue, the only mechanism of resistance the plant still has in its disposal is the hypersensitive response (HR), which is a specific type of programmed cell death [21,42]. Despite its irreversibility, it is a major defense mechanism against powdery mildew that has been described in numerous species and largely exploited in breeding for resistance against this pathogen [8].

Within several days of a successful infection, the development of the reproductive structures called conidiophores begins [12]. The first cell of the conidiophore becomes the so-called foot cell which supports one or more additional cells, including the apical generative cell that produces conidia. Conidia are single monokaryotic cells with a large number of water-filled vacuoles, which probably explains their unique ability to germinate in the absence of free water [43,44].

The exact mechanism of the release of conidia is unclear; the proposed mechanism involves mechanical force, wind and electrostatic charges [45,46,47]. The majority of the spores are released before midday, and a high relative humidity is known to impair this process [12,46,48]. The conidial dispersal is generally thought to occur over short distances, about 2 m from the host plant [49], though some findings, namely in the *Erysiphe* genus, suggest they can travel up to 700 km [50]. It appears that the amount of conidia produced and the density of the host population are directly related to the range of dispersal.

Sexual reproduction in powdery mildews occurs more rarely and is initiated by the production of male and female gametangia (antheridia and ascogonia, respectively) [51]. After the cytoplasmic merge (plasmogamy), the nucleus from the antheridium migrates into the ascogonium, resulting in the dikaryon [12,51]. Then monokaryotic cells at the base of the ascogonium begin to produce hyphae that form the outer layer of the ascocarp, which in powdery mildews is usually called the chasmothecium. The dykariotic ascogonium starts to divide, producing multiple dykariotic cells which then develop into asci that contain between two and eight ascospores, depending on the species [52]. Mature chasmothecia can be either anchored in place or separable from the parental mycelium [31,51,52]. In the second case, they have protrusions that could be helpful in dislodging them from the substrate and keeping them airborne as they are carried by the wind [12]. Sometimes ascospores can germinate within asci in the chasmothecium [53], but usually chasmothecium split open, discharging ascospores. The split is caused by the increased turgor pressure of the absorbed water, so the discharge occurs in high moisture, usually following rain [31,54].

Because Erysiphales are obligate parasites, they must be able to survive during periods when susceptible hosts are unavailable for infection. Chasmothecia are the primary dormant form of powdery mildew, which are suitable to withstand both cold winter and hot, dry summer [54]. Parts of mycelia with haustoria and even conidia can also persist within the dormant buds of host plants, causing a rapid infection shortly after sprouting [55].

### 2.2. Powdery Mildew in Legumes

The powdery-mildew disease in legumes provides an example of host–parasite specificity, although the exact taxonomic status of legume powdery mildews is still not well understood, as the taxonomy itself undergoes constant and rapid changes [56]. The only powdery-mildew species causing the disease in the majority of pulse crops, including peas (*Pisum sativum*) and alfalfa (*Medicago sativa* L.), was thought to be *Erysiphe pisi* DC, which consists of three *formae speciales* (host-specific groups): *E. p.*f.sp. *pisi*, *E. p.*f.sp. *medicaginis* and *E. p.*f.sp. *vicia-sativa* [57]. However, two more *Erysiphe* species have been reported to infect peas and lentils (*Lens culinaris* Medik.): *E. trifolii* Grev. and *E. baumleri* (Magnus) U. Braun & S. Takam [58,59,60]. The main agent of powdery mildew in soybeans (*Glycine max* (L.) Merr.) belongs to *E. diffusa* (Cooke & Peck) U. Braun & S. Takam (formerly *Microsphaera diffusa*), though *Erysiphe glycines* F.L. Tai has also been reported to infect this plant [8,61]. Cowpeas (*Vigna unguiculata* (L.) Walp.) and chickpeas (*Cicer arietinum* L.) are affected by *Podosphaera phaseoli* (Z.Y. Zhao) U. Braun & S. Takam and *Leveillula taurica* (Lév.) G. Arnaud, respectively [8]. Black gram (*Vigna mungo* (L.) Hepper) can be infected by *E. polygoni* DC [62].

The taxonomic ambiguity and difficulties with identification of powdery mildews are gradually becoming prominent obstacles to researchers and breeders, since even the strictest natural resistance is not universal and previously effective alleles could become useless against newly arisen pathogens. Meanwhile, powdery-mildew contamination can be fatal for legume crops. According to data, powdery-mildew outbreaks caused by *E. diffusa* can reduce the yield of soybeans by 50–60% in the cases of susceptible cultivars, with average losses being about 30% [63,64]. For the other legumes, the situation is not encouraging either: *E. polygoni* leads to a yield loss of black gram by 40% [65]; *E*. *pisi* can annihilate up to half (50%) of a pea crop [13,66]. Thus, strict pathogen control and breeding for new legume cultivars with broad resistance to powdery mildew are of paramount importance nowadays.

## 3. The Art of War

### 3.1. Chemical Control of the Powdery Mildew

Powdery mildew is one of the most prominent pathogens of legumes that is able to cause severe damage to the yield, thus a broad spectrum of disease-control methods is used to prevent its spreading. The most basic method is the appliance of various fungicides, mainly sulfur-containing compounds [13,67]. Different agents can affect fungus at different stages of the life cycle and in various physiological conditions, by disrupting cell division (benomyl, carbendazim), cell-wall synthesis and membrane functions (triadimenol, fenarimol, tridemorph) or spore germination (chlorothalonil) [68,69,70,71,72,73,74,75]. However, there are a number of serious drawbacks in this strategy. The cost and logistics of repeatable preventive appliances prevent many farm holds from using it, especially in the developing countries where the mildew problem is looming large. The application of fungicides only after the disease has already been observed is more realistic and cost-effective, but requires constant monitoring and precise timing, as the fungicide must be applied when the number of plants infected is low and the infection level of each plant is minimal [13]. Moreover, some of the most widely used fungicides have been prohibited in the EU since 2009 due to the environmental risks and/or human health issues they can cause (Regulation (EC) No. 1107/2009 of the European Parliament and of the Council). Despite all this, for lack of a better solution, fungicides continue to be widely studied and applied.

The negative public attitude towards the pesticides, environmental concerns, the expensiveness of commercial formulations, and the appearance of resistant powdery-mildew strains have led to the search for alternative methods to control the fungus. Non-fungicidal products used in powdery-mildew management include salts, oils, plant extracts, sclerotial exudates, compost tea extracts and even cow urine [76,77,78,79,80]. Some progress has been achieved using bergenin (isolated from *Flueggea microcarpa* Blume) [81], ajoene (constituent of garlic) [82] and extract from ginger [80,83]. Some of these agents are aimed at inducing or increasing the level of systemic resistance, thus “preparing” plants for subsequent pathogen attack. Plant hormones such as salicylic acid and pathogen-associated molecules such as chitosan are often used to that end with promising results [84,85]. This approach may potentially provide commercially useful broad-spectrum plant protection that is stable, readily available, long lasting and environmentally friendly.

### 3.2. Biological Control of the Powdery Mildew

As appealing as they may seem, the biological methods of powdery-mildew management remain a matter of the not-so-near future [13]. To date, there have been some encouraging results in the practical biocontrol of a number of powdery-mildew diseases, but they are mostly still limited to the laboratories and await large-scale agricultural trials. Attempts have been made to control powdery mildews with mycolytic bacteria, mycophagous arthropods, fungi, yeasts and other possible biological agents [86,87]. The most promising biological-control trials involving a number of natural antagonists of powdery mildews have resulted in the development of several biofungicide products. One well-known antagonist of species of the order *Erysiphales* and some other plant pathogens such as *Mucorales* and *Perisporales* is *Ampelomyces quisqualis* Ces. [87]. Considerable success was also achieved with *Trichoderma harzianum* Rifai, which resulted in a decreased number of colonies on the leaf surface [87,88]. Speaking of legumes, the importance of mutualistic plant–microbial interactions in terms of pathogen management should also not be neglected. A good example is the soybean interaction with *Bradyrhizobium japonicum* (Kirchner, 1896) Jordan, 1982. Nod factors (symbiotic signal molecules) of *B. japonicum* were reported to reduce the number of infection events and the size of powdery-mildew colonies [89]. A later study performed on peas (*Pisum sativum*) and barrel medics (*Medicago truncatula* Gaertn.) suggests that root symbiosis with rhizobia systemically primes plants for the salicylic-acid accumulation and defense-gene expression triggered by powdery mildew [90].

While the plant–rhizobial association is a specialized type of symbiosis that is inherent in a limited range of species, mycorrhiza is formed by approximately 90% of terrestrial plants [91]. Although mycorrhizal fungi are the most ancient mutualists of plants, the pattern of their behavior during host colonization strongly resembles that of the powdery mildews, which allows them to be considered as natural antagonists and potential biocontrol agents [92]. Indeed, it has been shown on multiple occasions that mycorrhiza positively affects the plant resistance to powdery mildew, decreasing the severity of infection [93,94,95]. However, given the wide variety of fungi species capable of forming mycorrhiza and the low specificity of this symbiosis, the data on the effectiveness of such a method of protection differ significantly depending on the specific host–symbiont pair. Thus, it was shown that wheat plants mycorrhized with *Funneliformis mosseae* (T.H. Nicolson & Gerd.) C. Walker & A. Schüssler exhibit resistance to the pathogen *Blumeria graminis* (DC.) Speer f. sp. *tritici*, reducing colonization by 78%, while association with *Rhizophagus irregularis* (Błaszk, Wubet, Renker & Buscot) C. Walker & A. Schüßler reduces colonization by only 34% [96]. At the same time, cucumber (*Cucumis sativus* L.) plants colonized by the arbuscular mycorrhiza fungus *Glomus intraradices* N.C. Schenck & G.S. Sm. did not demonstrate an increased resistance to powdery mildew [97], and standing milkvetch (*Astragalus adsurgens* Torr.) became even more susceptible to the disease when mycorrhized [98]. Taking all of the above into account, mycorrhiza undoubtedly benefits the plant, but it cannot be considered as the main and universal method of protection against powdery mildew.

Another microorganism suitable for powdery-mildew control is Bacillus subtilis, as well as *B. amyloliquefaciens*, *B. pumilus*, and several others [99,100,101,102,103,104,105]. Pseudomonas fluorescens and P. aeruginosa were also proven to suppress powdery mildew when applied on seeds as spray or suspension [105,106]. Recently, reports were published about the biocontrol potential of cyanobacteria (*Spirulina platensis* (Gomont) Geitler) in a mixture with P. fluorescens as well as on their own [107]. Non-pathogenic epiphytic microorganisms that normally colonize the plant can also play an important role in host defense against powdery mildew, either by competitive suppression, parasitism, antibiotic production or induced resistance [108,109]. *Pseudozyma flocculosa* (Traquair, Shaw & Jarvis) Boekhout & Traquair, the yeast-like basidiomycetous fungus, is unique in that it actually exploits the powdery-mildew pathogen (*B. graminis*) as a conduit to extract nutrients from the plant itself, causing the rapid disruption of mildew colonies [110]; nowadays, this fungus is considered one of the most promising biocontrol agents [109]. Nonetheless, most biological methods still need to be a part of an integrated treatment or a preventative measure, since the efficiency of bioagents can be significantly influenced by environmental conditions.

However, the most promising is the genetic approach based on the identification of naturally occurring/mutagen-induced variances with resistance to powdery mildew and their inclusion into breeding programs. Once established, genetic resistance is the cheapest and most efficient strategy of disease management, as it is more economically and environmentally friendly than, for example, chemical control. In the second half of the 20th century, the Eastern world turned to the ideas of humanism and began looking for solutions to the global problems of humanity, in particular, the problem of starvation, which was especially acute in developing countries. Legumes are excellent sources of nutrition, which, together with them being undemanding in terms of soil conditions due to nitrogen fixation, makes them an essential component of agriculture in these countries [111,112]. Unfortunately, the range of crops available in these countries is usually limited to the so-called “landraces” with low productivity, and the introduction of quality cultivars is complicated by high pathogenic activity, especially with regard to fungi [113,114]. To improve the quality of cultivars, about 50 years ago a worldwide program was started with the aim of the improvement of crops in terms of yield stability and performance, with much attention focused on disease resistance (World Food Conference, 1974; World Conference on Agrarian Reform and Rural Development, 1979). Among legumes, special attention was paid to mung beans, soybeans, cowpeas, chickpeas and garden peas, for which resistance to powdery mildew has become one of the fundamental selective traits, since this disease is especially dangerous in the regions of their active cultivation. Since then, researchers have made remarkable strides in finding disease-resistant legume varieties, identifying the genes responsible for this trait, and using this knowledge in breeding programs. However, there are certain difficulties and nuances that will be discussed below.

## 4. Close the Gate

When it comes to the genetic mechanisms of resistance to pathogens, the key role belongs to the so-called systemic acquired resistance (SAR), which is associated with the expression of resistance genes (R-genes) and the triggering of the hypersensitivity reaction (HR) [9,115,116]. This mechanism allows plants to cope with the daily presence of nonspecific and opportunistic pathogens. On the other hand, specific pathogens, for which a given plant is the primary host, have evolutionarily acquired ways to bypass the SAR by interacting with special target proteins, which serve as a kind of “gateway” into the host organism [116,117]. The genes encoding such proteins are called S-genes (susceptibility genes). The loss-of-function mutations in S-genes often lead to the broad-spectrum resistance to a particular kind of pathogen, as they effectively “close the gates” to the plant for this pathogen [115,116].

The history of studying the genetic mechanisms of plant resistance to powdery mildew began with barley (*Hordeum vulgare* L.), which was found to have the *Mla* gene locus (for “Mildew locus A”) that provides resistance to *Blumeria graminis* f. sp. *hordei* based on the “gene-for-gene” principle [118,119]. The *Mla* genes (*Mla-1* to *Mla-32*) are typical R-genes that encode the nucleotide-binding-site LRR proteins, presumably allowing recognition of race-specific fungal proteins, which leads to the induction of HR and the development of SAR [120,121]. Nonetheless, due to their nature, *Mla* genes are only able to provide race-specific resistance to powdery mildew. However, durable broad-spectrum resistance to *B. graminis* f. sp. *hordei* in barley also exists, caused by recessive, loss-of-function mutations in a single gene called *Mlo* (for “Mildew locus O”) [122].

*Mlo* encodes an integral-membrane protein with seven transmembrane helices and is a classic S-gene that allows fungi to penetrate plant cells [123,124]. Mutations that disrupt its structure prevent the normal fungus penetration through the epidermal cell wall without triggering a cell-death response. Further studies revealed that *MLO* genes (note that there are differences in nomenclature depending on the species: in barley, they are traditionally written as *Mlo*, while *MLO* is used as a more general spelling) occur as small to medium-sized families in the genomes of higher plant species, monocots and dicots alike [124,125]. This discovery led to the identification of natural or induced loss-of-function *mlo* alleles associated with powdery-mildew resistance in several agriculturally important plants, including the garden pea, which is the representative of the legumes [14,15]. It appeared that MLO proteins were part of a sizable family. The 15 *Arabidopsis* MLO proteins were originally grouped into four major clades (I-IV) [126]. Subsequently, a more detailed analysis including 17 MLO members of the grapevine (*Vitis vinifera* L.) and several of those from barley, bread wheat (*Triticum aestivum* L.), rice (*Oryza sativa* L.) and corn (*Zea mays* L.) distinguished six distinct clades [127]. Additionally, several studies have proposed the existence of clade VII, which would be represented by MLO11 from the cucumber (*Cucumis sativus* L.) [128] and MLO2 from the tomato (*Solanum lycopersicum* L.) [129]. The recent study of [130] expanded clade VII to 21 members from dicots, as well as postulated the existence of clade VIII with six monocot-specific members. The work is still in progress, and the data are constantly being updated. To date, all MLO proteins from dicot species that are known to be associated with powdery-mildew susceptibility are sorted into clade V, while the monocot species are grouped into clade IV along with several recently discovered representatives from the dicots, namely legumes (*Medicago truncatula*) [124,127,131].

The exploitation of such an appealing trait as *mlo*-based resistance in a broad spectrum of crops is not without its limitations. It appears that, in the absence of pathogens, barley *mlo* mutants tend to spontaneously form callose-containing cell-wall papillae, predominantly in the short-cell type of the leaf epidermis [132]. Additionally, leaf mesophyll cells in *mlo* mutants undergo spontaneous cell death [133], which has been recognized as an indication of accelerated leaf senescence. Similar *mlo*-associated pleiotropic effects have also been observed in *Arabidopsis* plants [38,134].

Being the first legume species with well-documented *mlo*-based resistance (caused by the loss of function in gene *MLO1*, also known as *Er1* [14,15]), the garden pea (*Pisum sativum*) possesses yet another notable feature: known pea mlo mutants are spared the negative pleiotropic effects of their mutations [14]. It is thus no wonder that from the moment of its discovery to this day, *MLO1* remains a very actively studied gene. There are eleven previously reported *Psmlo1* (*er1*) alleles, either of natural origin or obtained by mutagenesis, that confer resistance to the main legume powdery mildew *Erysiphe pisi* (see Table 1). Several function markers covering the majority of these alleles have been designed for breeding assistance [15,135,136,137,138,139,140]. The *er1-7* and *er1-8* alleles were independently found in the analysis of powdery-mildew-resistant accessions from the pea-germplasm collection of the John Innes Centre (Norwich, UK) performed in the All-Russia Research Institute for Agricultural Microbiology (Saint-Petersburg, Russia). The sources of these alleles have germplasm collection numbers JI1128 and JI1171 (*er1-7*) and JI0092, JI0101 and JI0105 (*er1-8*). In the same analysis, a new resistant allele *er1-12* with an A insertion at position 1735 of the *PsMLO1* cDNA was also identified. The line carrying this allele is named JI2019 in the John Innes Centre germplasm collection and originates from India [141]. The details of this work are described in Appendix A.

## 5. Gathering Forces

The successes achieved with peas, coupled with advances in molecular biology and the development of “omics” technologies, stimulate the search for *mlo*-based immunity in other legumes, from well-established models such as the barrel medic to valuable crops such as soybeans to so-called “orphan” crops such as mung beans or chickpeas [21,131,146,147,148,149]. A promising approach is a whole-genome screening for all the sequences homologous to *MLO* with subsequent targeted knockout using CRISPR-Cas, or a search for induced mutants with TILLING [150]. For some species, there is previously obtained data on resistance to powdery mildew available, including the most prominent model legume, the barrel medic (*Medicago truncatula*) [21]. With its diploid and relatively small (~500 Mb) genome, self-pollination and close relations with main-crop legumes, *M. truncatula* is an extremely convenient object of study. The fact that *M. truncatula* can serve as a resource of resistance genes to powdery mildew is evidenced by various phenotypes, from completely resistant to highly susceptible [151,152]. This wide range of plant reactions to powdery mildew suggests the existence of different resistance mechanisms which, given the development level of *M. truncatula* genetics, could be thoroughly investigated. Unfortunately, the amount of resistant *M. truncatula* cultivars discovered so far is not large; for instance, of 277 samples, only 10 showed complete or moderate sustainability [151].

In 2008, three major QTLs of *M. truncatula* responsible for different levels of powdery-mildew resistance were found, namely *Epp1* (located on chromosome 4), *Epa1* and *Epa2* (both located on chromosome 5) [153]. It is worth mentioning that the phenotype of different genetic mechanisms of resistance was the same: *E. pisi* was not able to penetrate the cell wall of the plant (which suggests the possible involvement of *MLO* homologs). In 2016, a wide search for *MLO*-like sequences was performed among the seven legume species with available genomes, namely *M. truncatula*, *Cicer arietinum*, *Lupinus angustifolius* L., *Cajanus cajan* (L.) Huth, *Phaseolus vulgaris* L., *Vigna radiate* (L.) R. Wilczek and *Arachis* spp. [131]. The transcriptomic data for *Pisum sativum* were also included. All studied genomes contained between 14 and 23 sequences with homology to *MLOs* from *Arabidopsis thaliana* used as a BLAST query, including some short truncated sequences. Most of the latter were designated as inactive pseudogenes due to their proximity to the retrotransposon-like sequences. However, some of these short genes probably belong to a clade VII of *MLO*, as similar sequences were detected in the cucumber, tomato, apple and strawberry. The exact properties and functions of these genes remain to be studied.

In *M. truncatula*, 16 *MLO* genes were found, including two dubious truncated sequences. The 14 “true” *MLOs* were distributed among 6 of the 7 *MLO* clades. However, the most intriguing is the fact that besides two clade V *MLO* genes usually found in temperate legumes, *M. truncatula* also possessed one clade IV *MLO* gene that was thought to be restricted to monocots. The function of this additional gene in eudicots is currently unknown.

Not only the genetic aspects can be studied in the *E. pisi-M. truncatula* pathosystem; the role of epigenetics is also of high interest. In 2013, Yang et al. [154] found the *MtREP1* gene on chromosome 5. Three candidate genes for its role were identified, but only one remained—*Medtr5g072340*—because plants carrying this gene showed resistant phenotypes. Further studies revealed that the key difference between dominant and recessive alleles was not in the sequence itself, but in the epigenetic modification, specifically in different levels of methylation.

The soybean (*Glycine max*) is one of the world’s most important protein sources and the most cultivated legume. At least three species from the *Erysiphe* genus are reported to create a pathosystem with the soybean: *E. pisi* (pathogenic for a wide range of legumes), *E. diffusa* and *E. glycines* [61]. It is known that soybean resistance to *E. diffusa* is regulated by three alleles of the *Rmd* locus, located on chromosome 16 (linkage group J) [155,156]. *Rmd-c* is required for complete resistance to powdery mildew throughout the whole life cycle of a plant. *Rmd* causes resistance only at late stages of development, while *rmd* is found in susceptible plant samples [157]. In addition, another gene of resistance was shown, namely *Rmd_PI243540*, which also provides resistance throughout all stages of growth. In this case, the trait is controlled by a single dominant gene [63,158].

The soybean was actually the very first legume to undergo a wide-scale search for *MLO* homologues [148]. Subsequently, several *MLO* genes were found located on the same chromosome where *Rmd* was previously mapped. Overall, 31 full-length *MLO* sequences were identified, making the soybean one of the most, if not the richest legume in terms of *MLO* genes. These genes were present on 15 of 20 chromosomes, mostly located on chromosome 12 (5 *MLO* genes). *G. max* is known as a paleopolyploid that underwent a couple of duplication events millions of years ago [159,160], which could explain the high number of *MLO* in its genome.

Later, two more representatives of the Milletoid clade, the pigeon pea (*Cajanus cajan*) and common bean (*Phaseolus vulgaris*) [146,161], have been screened for *MLO*-like sequences. A total of 18 (*C. cajan*) and 20 (*P. vulgaris*) *MLO* family members have been identified, and for each species, two genes have been predicted to participate in powdery-mildew resistance. So far, similar work has been performed on lentils (*Lens culinaris*) [162], mung beans (*Vigna radiata*) [163], cultivated peanuts (*Arachis hypogaea* L.) [164], as well as many non-legume species. In each case, candidates have been identified for the role of powdery-mildew-resistance genes.

## 6. Para Bellum: Future Perspectives and Challenges

Powdery-mildew control remains an important problem for modern agriculture, despite the incremental growth in the knowledge of the pathogen itself and the molecular mechanisms of plants’ defense against it. In accordance with new findings, the systematics of pathogenic fungi is undergoing significant changes, but only a fraction of the described species have been reassessed using molecular-genetics approaches and some large areas still rely on the obsolete taxonomy [12]. Moreover, some mildew species seem to rapidly increase their geographical distribution, presumably due to climate changes, bringing disturbances and changes into long-established host–pathogen systems [12,165,166]. In legumes, this is reflected by the fact that new pathogens are being found that were unknown to cause disease in a given species. In particular, *Erysiphe baeumleri* and *Erysiphe trifolii* have recently been reported in peas and lentils that were previously thought to be damaged only by *E. pisi* [58,59]. As a result, we are becoming increasingly aware that even the most reliable methods of dealing with mildew can misfire when the pathogen’s disposition changes unpredictably. Under these circumstances, a promising approach seems to be not to endow cultivars with resistance to pathogens by introducing R-genes, which eventually could be overcome by pathogens that manage to win the “Red Queen race” (see the Red Queen hypothesis, according to which, if a species is to survive, it must evolve continuously and rapidly [167]), but to modify the systems used by pathogens to colonize the host (S-genes) so they can no longer serve this purpose. This will result in broad and durable resistance to pathogens, as seen in the case of powdery mildew [115,116].

Among all of the agriculturally important legumes, the pea can benefit the most from S-gene-based broad-spectrum resistance to powdery mildew, since it demonstrates no negative side effects of loss-of-function mutations in the *MLO1* gene, also known as *Er1*. This is probably the reason for the presence of such a large number of naturally occurring resistant alleles of *MLO1* in pea germplasm; at the moment, 11 of them have been described, including one found by the authors of this review (Table 1). Moreover, it has two more described genes conferring powdery-mildew resistance, recessive *er2* and dominant *Er3*, which act at different stages of the fungus penetration [35,168]; additional genes are being thoroughly researched using the genomic assembly that was recently made available and modern genome-wide methods of analysis [169,170]. The data obtained on other legume species, such as *Medicago truncatula* or *Glycine max*, have also expanded the knowledge about the diversity of the molecular mechanisms of resistance of legumes to powdery mildew. Since legumes are important components of modern agronomy due to their ability to fix atmospheric nitrogen in symbiosis with nodule bacteria, this knowledge will be of high demand for the creation of new varieties of legumes that are resistant to powdery mildew, for instance, by using genome-editing technologies for modification of the host S-genes, similarly to the naturally-occurring *mlo1*-based system in the pea.

## Figures and Tables

**Table 1:** List of known *Psmlo1* (*er1*) alleles conferring resistance to powdery mildew.

*Psmlo1* Allele	Mutation	Cultivar/Landrace	Place of Origin
*er1-1* *(er1mut1)*	C → G at position 680 of cDNA	Mexique 4/JI1559 [14]	Mexico
		S [142]	Portugal (mutant obtained on the Solara cultivar)
		Tara [143]	Canada (obtained on material from Argentine)
*er1-2*	Large transposon insertion of unknown size in 14th exon (position 1262 of cDNA) leading to formation of various aberrant *MLO1* transcripts	Stratagem/JI2302 [14]	Mexico
		Franklin/PI 628275; Dorian; Nadir [15]	USA (on an unknown material)
		X9002 [137,144]; Xucai 1 [145]; G0005576 [144]	China
*er1-3*	G deletion at position 862 of cDNA	JI0210/Lucknow Boniya/PI 240515 [14]	India
*er1-4*	A deletion at position 91 of cDNA	YI; JI1951 [14]	China
*er1-5*	G → A at position 567 of cDNA	ROI3/02 [15]	Italy (mutant obtained on the Sprinter cultivar)
*er1-6*	T → C at position 1121 of cDNA	Wandou G0001752, Baiwandou G0001763, Dabaiwandou G0001764, Fanwandou G0001767, Wandou G0001768, Dabaiwandou G0001778, Dabaiwandou G0001780, Wandou G0003824 [144]	China
*er1-7*	TCATGTTATT deletion at position 111–120 of cDNA	DDR-11 [136]	India
		JI1128 (see Appendix A of this article)	India
		JI1171 (see Appendix A of this article)	India
*er1-8*	GTG deletion at position 1339–1341 of cDNA	G0004839 [135]	Afghanistan
		JI0092/PI 134271 (see Appendix A of this article)	Afghanistan
		JI0101/PI 220175 (see Appendix A of this article)	Afghanistan
		JI0105/PI 222070 (see Appendix A of this article)	Afghanistan
*er1-9*	T deletion at position 928 of cDNA	G0004400 [135]	Australia
*er1-10 (er1mut2)*	G → A at position 939 of cDNA	F [142]	Portugal (mutant obtained on the Frilene cultivar)
*er1-11*	GT insertion at position 2974–2975 of genomic DNA (intron 11)	Yarrum, ps1771 [138]	Australia
*er1-12*	A insertion at position 1735 of cDNA	JI2019 (see Appendix A of this article)	India

**Figure A1:**
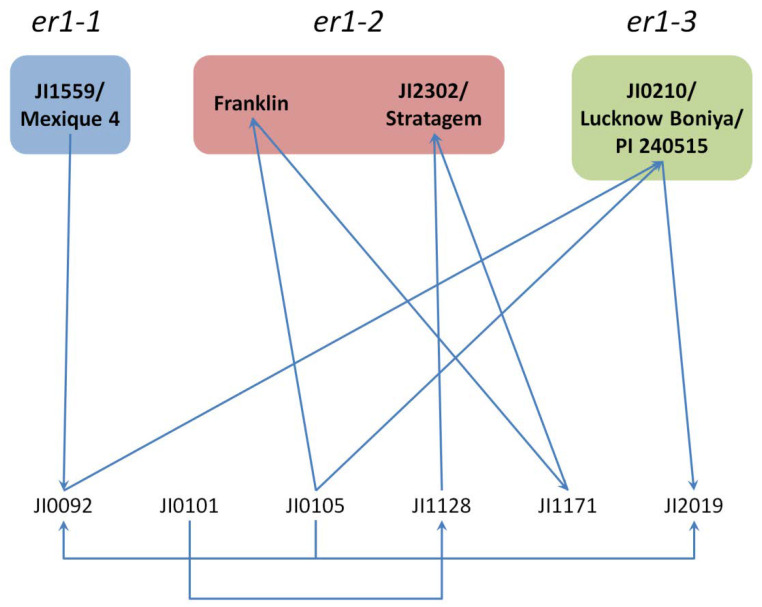
Scheme of the allelism test performed with the pea genotypes from the John Innes *Pisum* germplasm collection with unknown genetic determinants of resistance to powdery mildew (**bottom row**) and genotypes with previously described resistant *MLO1* alleles (**top row**). Arrows indicate directions of crosses.

**Figure A2:**
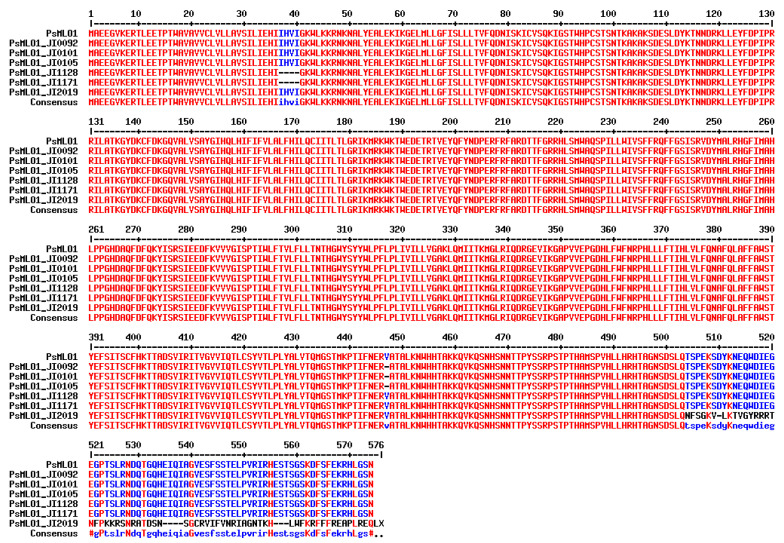
Comparison of translated protein sequences of MLO1 (Er1) of studied pea genotypes (obtained via Multalin web service [173], http://multalin.toulouse.inra.fr/multalin/multalin.html).

## Appendix A

Samples from the John Innes *Pisum* germplasm collection were selected based on the results of field screening for powdery-mildew resistance [141]. The selected samples were first subjected to the allelism test with several previously described *er1* pea lines (see Figure A1). For each crossing, at least five hybrid plants were tested. Hybrids were inoculated with powdery mildew by brushing seedlings with an infected pea plant. The phenotype was evaluated visually two weeks after inoculation by the presence/absence of mildew spots on leaves. All hybrids proved to be resistant to powdery mildew, thus the determinant of their resistance is allelic to *Er1*(*MLO1*).

For the molecular-genetic analysis, plant DNA was extracted from young leaves (top or second-from-top node) according to the previously described CTAB protocol [171,172]. RNA was extracted from similar material that was pre-frozen in liquid nitrogen and ground with a pestle and mortar. The isolation procedure was performed using the NucleoSpin miRNA kit (Macherey-Nagel, Düren, Germany) according to the protocol recommended by the manufacturer. The isolated RNA was additionally treated with DNase (RQ1 DNAse, Promega, Madison, WI, USA) to remove DNA residues from the samples. The concentration of the obtained RNA was determined on a Qubit 3 fluorometer (Thermo Fisher Scientific, Waltham, MA, USA). The first strand of cDNA was synthesized on a total-RNA template using the Mint cDNA kit (Evrogen, Moscow, Russia) according to the manufacturer’s protocol.

The PCRs were performed in 0.5 mL Eppendorf-type microcentrifuge tubes on an iCycler (Bio-Rad, Hercules, CA, USA) or Dyad (Bio-Rad, USA) thermocycler using the ScreenMix-HS kit (Evrogen, Moscow, Russia). The cycling conditions were as follows: 95 °C (5 min), 35 × [95 °C (30 s), Tm (varying depending on primers) (30 s), 72 °C (1 min)], 72 °C (5 min). The PCR fragments were sequenced using the ABI Prism3500xL system (Applied Biosystems, Palo Alto, CA, USA) at the “Genomic Technologies, Proteomics, and Cell Biology” Core Center of the ARRIAM (St. Petersburg, Russia). Primers used in this work are listed in Table A1.

**Table A1:** Primers used in PCR analysis.

Name	Sequence, 5′–3′	Melting t°
Er1_fw0	GAA AGA AAA AAT GGC TGA AGA GG	59.2
Er1_fw1	GAT AAG GGT CAA GTT GCA TTA G	58.4
Er1_fw3	TTT CCA AAA GTA TAT AAG TAG ATC	55.0
Er1_rv2	TAA GAA GGA AAA GCA CTG TGA AG	59.2
PsMLO7Fw	ATG CCA TGT CTC CTG TTC ACC	61.2
PsMLO5Rv	CTT TAT CTG CAA GAA TGT ACC	55.4

The same primers were used for both cDNA and gDNA amplification, as they are positioned within exons.

Of six lines tested, five proved to possess already described *er1* alleles, namely *er1-7* (JI1128 and JI1171) and *er1-8* (JI0092, JI0101 and JI0105). However, line JI2019 appeared to have previously unknown *er1* allele with an additional A in the beginning of the last exon which shifts the reading frame (see Figure A2). Thus, we propose the name *er1-12* for this newly described *PsMLO1* allelic variant.
